# Spatiotemporal Interpolation for Environmental Modelling

**DOI:** 10.3390/s16081245

**Published:** 2016-08-06

**Authors:** Ferry Susanto, Paulo de Souza, Jing He

**Affiliations:** 1 Data61, CSIRO, College Road, Sandy Bay TAS 7005, Australia; Paulo.desouza@data61.csiro.au; 2 College of Engineering and Science, Victoria University, Footscray VIC 3011, Australia; Jing.He@vu.edu.au

**Keywords:** spatiotemporal interpolation, ordinary kriging, inverse distance weighting, triangular irregular network, distribution-based distance weighting

## Abstract

A variation of the reduction-based approach to spatiotemporal interpolation (STI), in which time is treated independently from the spatial dimensions, is proposed in this paper. We reviewed and compared three widely-used spatial interpolation techniques: ordinary kriging, inverse distance weighting and the triangular irregular network. We also proposed a new distribution-based distance weighting (DDW) spatial interpolation method. In this study, we utilised one year of Tasmania’s South Esk Hydrology model developed by CSIRO. Root mean squared error statistical methods were performed for performance evaluations. Our results show that the proposed reduction approach is superior to the extension approach to STI. However, the proposed DDW provides little benefit compared to the conventional inverse distance weighting (IDW) method. We suggest that the improved IDW technique, with the reduction approach used for the temporal dimension, is the optimal combination for large-scale spatiotemporal interpolation within environmental modelling applications.

## 1. Introduction

Environmental sensor networks offer a significant contribution to our society, and the data collected are crucial for environmental managers to support decisions for the effective use of natural resources. Applications include environmental monitoring for hazard warning services (e.g., cyclone, flood and bushfire) [1], which introduces the need for environmental scientists to have spatially- and temporally-continuous data flows to support accurate and justified interpretations [2].

However, because of current technology limitations, we are not yet able to obtain high quality data in terms of spatial and temporal coverage [3], especially in mountainous and deep marine regions. The main restriction is the cost to set up comprehensive sensor networks in these environments and in a real-time manner. It is arguable whether it is necessary to set up such costly networks to obtain high quality data; while we could set up the network only at some critical points and apply appropriate interpolation technique to estimate the remaining unmeasured locations.

Spatial interpolation studies have been conducted in many environmental projects, involving rainfall, temperature and air pollution data. Table 1 lists some of the reviewed papers that make use of different interpolation methods for different purposes (Table 2 provides the list of interpolation abbreviations used within the table). Li and Heap [2] have provided a comprehensive review of the most commonly-used spatial interpolation techniques used in different areas: environment, hydrology, agriculture, forestry and engineering. A list of statistical error measurements to evaluate the interpolation model are provided in their work.

Based on the findings in Table 1, kriging has been reported as the most suitable spatial interpolation method overall [8,10,11], yet it suffers from the drawback that it is very computationally demanding. Subsequently, IDW has been extensively used and improved in many studies because of its advantages of efficiency and comparable performance with kriging [4,6,7]. Sanabria et al. have shown that using a hybrid method (RFOK and RFIDW) can enhance the quality of the interpolator [1].

None of the methods is uniquely optimal for any particular situation [12,13]. These factors are described in detail by Li and Heap [2]: (a) the sampling design and the samples’ distribution; (b) the data nature and quality; (c) the correlation between primary and secondary variables. Therefore, the best technique for a specific purpose can only be obtained by comparing them and selecting the one that is most appropriate for the particular task [3,9,11].

Nevertheless, not much research has been reported on interpolation methods that include the temporal dimensionl: spatiotemporal interpolation (STI) techniques. Li proposed using triangular meshes to perform spatiotemporal interpolation to measure the housing prices in Lincoln, Nebraska [14]. Then, a triangulation-based STI method was applied to the air pollution study as reported in 2011 [15]. Later in 2014, Li and collaborators utilised an IDW-based STI to further improve interpolation performance by increasing computational efficiency [16].

Kilibarda and collaborators utilised a geostatistical STI method to model global surface temperature [17]. They indicate that this kind of STI method also facilitates computational efficiency by creating a single semivariogram for the entire year, instead of 365 semivariograms for each day in a year. Such an interpolator does not show abrupt jumps between days that have no physical basis.

### 1.1. Objective

In this study, we propose a new spatiotemporal interpolation (STI) technique with the following features:

1. Computationally effective: The sensor network is assumed to collect hundreds (or even thousands) of data items every second, and thus, a fast algorithm is critical for effective processing.
2. The ability to obtain close-to-reality measurements (i.e., low statistical error and visually not-abrupt: To ensure environmental managers can make confident interpretations at a later stage.

## 2. Materials and Methods

### 2.1. Experimental Data

**Figure 1:**
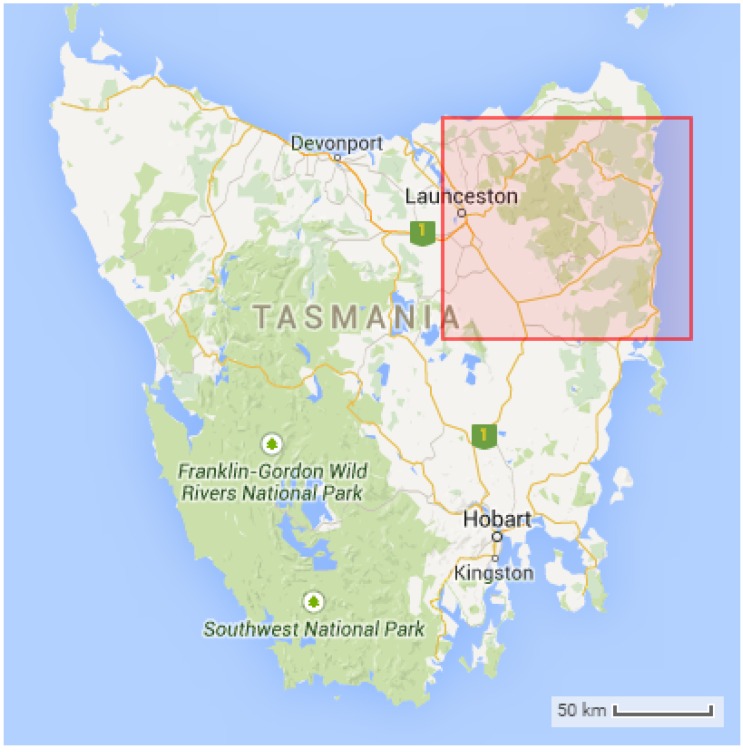
Location of South Esk Hydrological model, northeast of Tasmania, Australia. The exact coordinate locations of the red bounded region are: 42°00${}^{\prime }$S, 147°00${}^{\prime }$E (bottom, left) and 41°00${}^{\prime }$S, 148°30${}^{\prime }$E (top, right), which covers approximately 18,588 km${}^{2}$ (image adapted from Google Maps).

The dataset used in our experiment is the Tasmania’s South Esk Hydrological dataset (Figure 1), developed by CSIRO [18]. It is in netCDF format, a common data format used for environmental monitoring, in which data are stored within multi-dimensional gridded arrays.

In this dataset, the map is gridded every 0.01 degree (≈1.1 km${}^{2}$ spatial area), which results in 151 horizontal and 101 vertical grids (a total of 15,251 cells). The data also have a temporal dimension that records data hourly within the area of interest.

There are a number of environmental parameters available within the dataset, such as temperature, relative humidity, solar radiation, surface height, and so on. In this paper, we used the surface height data to select the near optimal location of sensor nodes for determining the most appropriate spatial interpolation technique, and such a selection will be justified in Section 3.1. Accordingly, we adopt the air temperature parameter data for the whole year of 2013, from 5 January to 27 December (inclusive) for the rest of the simulation. However, the dataset still contains some gaps, and these will not be included in the simulation process to ensure the integrity of the final results.

### 2.2. Interpolation Techniques

#### 2.2.1. Ordinary Kriging

In this paper, we utilised OK as the kriging-based interpolator because it is the most-widely used kriging method for interpolation. Furthermore, based on the fact that we will compare techniques based on geostatistical and non-geostatistical methods, for the case of fairness, we only consider kriging-based methods that are “univariate” [2].

OK, also called the OK estimator, is categorised as a geostatistical method; it takes spatial patterns and the uncertainty of the surface into account during the interpolation process [13]. A semivariogram is calculated to show how much a point in space is related to the points around it within a particular distance (neighbouring values) using the following equation:

(1) $$\hat{\gamma }\left(h\right)=\frac{1}{2n}\sum_{i=1}^{n}{\left[z\left(x_{i}\right)-z\left(x_{i}+h\right)\right]}^{2}$$

where *n* is the number of pairs of observed points *z* that are separated by distance *h*. Subsequently, the $\hat{\gamma }\left(h\right)$ is plotted against *h* and is called the experimental variogram. The variogram curve needs to be fitted for the OK estimator; the spherical model is the most commonly used and it will also be utilised in this work. The conventional kriging estimator is of the form:

(2) $$\hat{z}\left(x_{0}\right)=\sum_{i=1}^{n}\lambda_{i}\left[z\left(x_{i}\right)\right]$$

where $\hat{z}\left(x_{0}\right)$ is the interpolation value at any unmeasured location; $\lambda_{i}$ is the kriging weight measured at the *i*-th point with $\sum_{i}^{n}\lambda_{i}=1$; with the calculation based on minimising the variance of $\hat{z}$:

(3) $${\hat{\sigma }}^{2}=\sum_{i=1}^{n}\lambda_{i}\gamma \left(x_{i},x_{0}\right)+\phi$$

where *φ* is the Lagrange multiplier, which is required for the minimisation.

#### 2.2.2. Shape Function: Triangular Irregular Network

TIN is a digital means of representing surface morphology and has been extensively used in the GIS community. It consists of connecting edges between vertices that eventually form a network of triangles, which is normally constructed using the Delaunay triangulation algorithm.

**Figure 2:**
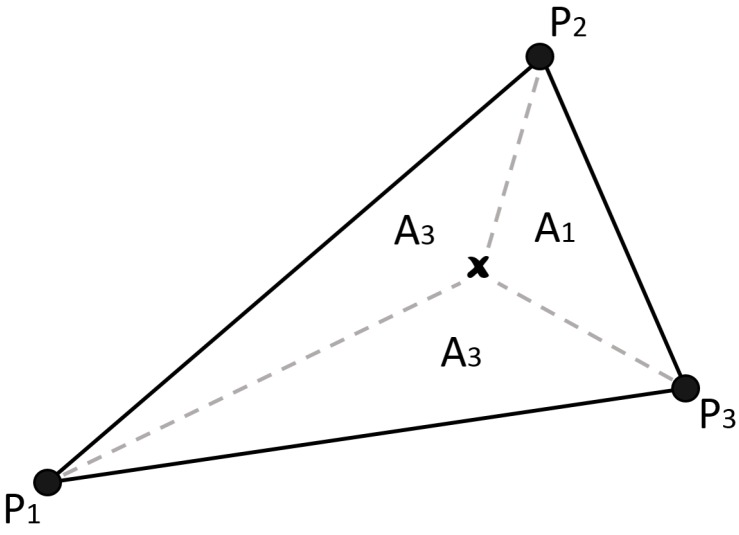
An illustration of the TIN interpolation technique at point *x*. In this case, point *w* lies within a triangle formed by points $P_{1}$, $P_{2}$ and $P_{3}$. The weight of each point is calculated based on the corresponding area. For example, $P_{1}$ has the weight of $\frac{A_{1}}{\sum A_{i}}$, and so on.

This surface analysis technique can be further extended as a linear approximation interpolation algorithm as proposed by Peuker and co-workers in 1978 for digital elevation modelling [12]. It was described in the work done by Li in 2003, which uses area divisions for the weighting mechanism [14] (Figure 2). As it is based on triangle meshes, the total number of included observed points is 3. It is in the form of:

(4) $$\hat{z}\left(x_{0}\right)=\sum_{i=1}^{N=3}\lambda_{i}\cdot z\left(x_{i}\right)$$

(5) $$\lambda_{i}=\frac{A_{i}}{\sum_{i=1}^{N=3}A_{i}}$$

(6) $$A_{i}=\frac{1}{2}det\left[\begin{array}{ccc} 1 & x_{1} & y_{1}\\ 1 & x_{2} & y_{2}\\ 1 & x_{3} & y_{3} \end{array}\right]$$

where *A* is the area of the entire triangle and $A_{i}$ is the *i*-th sub-triangle formed by the point to be interpolated (*x* in Figure 2).

#### 2.2.3. Classic Inverse Distance Weighting

The traditional IDW interpolation technique proposed by Shepard in 1968 [19] uses the following equations:

(7) $$\hat{z}\left(x_{0}\right)=\sum_{i=1}^{N}\lambda {\left(d_{s_{i}}\right)}_{i}\cdot v_{i}$$

(8) $$\lambda {\left(d_{s_{i}}\right)}_{i}=\frac{d_{s_{i}}^{-u_{s}}}{\sum_{i}^{N}d_{s_{i}}^{-u_{s}}}$$

(9) $$d_{s_{i}}=\sqrt{{\left(x_{i}-x\right)}^{2}+{\left(y_{i}-y\right)}^{2}}$$

where $d_{s_{i}}$ is the 2D-spatial Euclidean distance between the *i*-th known data point and the unmeasured location point; $\lambda (d_{s_{i}})$ and $v_{i}$ are the weights and values assigned to each of the observed points; and $u_{s}$ is the spatial distance factor parameter specified by the user.

#### 2.2.4. Improved Inverse Distance Weighting

**Figure 3:**
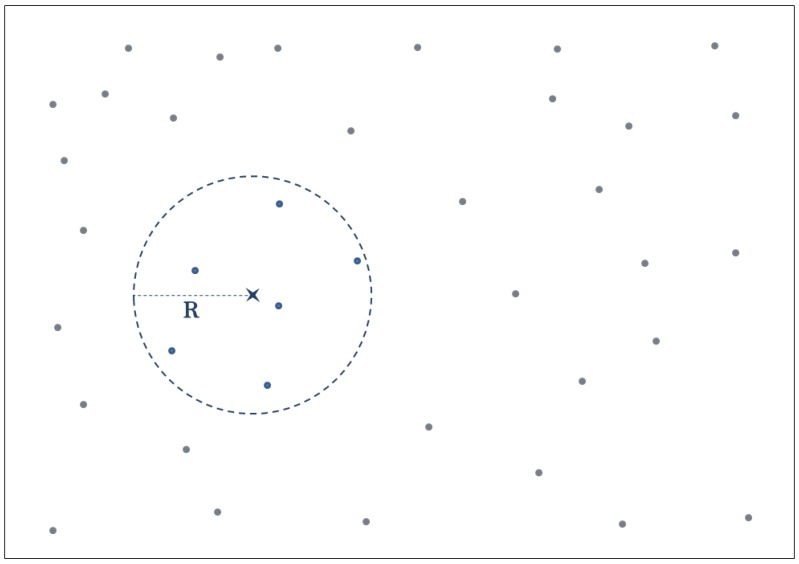
A demonstration of the improved IDW. The dots are the sample data points, and the *x* is the point location to be interpolated. R is a user-defined radius parameter that indicates the farthest distance to be included from the points *x*. In this case, only a total of six sample points will have an influence on the interpolation process. However, no empirical approach has been developed to obtain the optimal value for the parameter *R*.

IDW is based on the notion that nearer data points will have more influence than those further away, and so, including every data point throughout the map to interpolate a single point is unnecessary. This is because as the distance is further away; the particular point has very little influence on the final value. An illustration of the improved version of IDW can be seen in Figure 3.

On the other hand, de Mesnard has discussed the problem of 0 distance in IDW (i.e., $d_{s_{i}}=0$ as in Equation (9)), and it is divided into two categories, such that there is a discrete and a continuous case [20]. In this paper, since our dataset is based on the gridded system (Section 2.1), it implies that the value within a single cell is actually the mean value of the entire 1.1 km${}^{2}$ area. Therefore, we can justify that our dataset lies within the discrete category and that we can simply obtain the value at the sample point when the algorithm tries to interpolate a reference point.

The improved IDW comes with two major advantages in terms of computational efficiency: (a) the processing time does not increase as the number of known points increase; (b) we can further improve the performance by applying the kd-tree data structure algorithm, reducing the computational time from O(*n*) to O(logn) [16]. Furthermore, this feature is crucial because the basic computational time required for the STI algorithm is O(t*n), instead of O(*n*), where *t* is the number of user-defined window lengths to be included in the process.

#### 2.2.5. STI Technique : Extension Approach

The extension approach to the IDW spatiotemporal interpolation method is described in [16]:

(10) $$\hat{z}\left(x_{0},t\right)=\sum_{t_{start}}^{t_{end}}\sum_{i=1}^{N}\lambda {\left(d_{st_{i}}\right)}_{t,i}\cdot v_{t,i}$$

(11) $$\lambda {\left(d_{st_{i}}\right)}_{t,i}=\frac{d_{st_{i}}^{-u_{s}}}{\sum_{i}^{N}d_{st_{i}}^{-u_{s}}}$$

(12) $$d_{st_{i}}=\sqrt{{\left(x_{i}-x\right)}^{2}+{\left(y_{i}-y\right)}^{2}+c^{2}{\left(t_{i}-t\right)}^{2}}$$

where $d_{st_{i}}$ is the spatiotemporal distance between the measured $\left(x_{i},y_{i},ct_{i}\right)$ and unmeasured (x,y,ct) location point, and cis the user-defined temporal factor. However, there is still no empirical information on how to justify the temporal factor (*c*), and a naive choice does not yield optimal results [16]. 

### 2.3. Proposed Methodology

#### 2.3.1. STI Technique: Reduction Approach

The main contribution in this paper is to propose another variation of the IDW-based STI algorithm, namely the reduction approach, which treats time independently from the spatial distance dimension [14]. Based on the conventional IDW technique, this practice calculates the weight in two steps: firstly using the inverse of 2D-spatial distance, followed by the inverse of the 1D-temporal distance. As below:

(13) $$\hat{z}\left(x_{0},t\right)=\sum_{t_{start}}^{t_{end}}\sum_{i=1}^{N}\lambda {\left(d_{s_{i}},d_{t_{i}}\right)}_{t,i}\cdot v_{t,i}$$

(14) $$\lambda {\left(d_{s_{i}},d_{t_{i}}\right)}_{t,i}=\frac{d_{s_{i}}^{-u_{s}}\cdot d_{t_{i}}^{-u_{t}}}{\sum_{i}^{N}d_{s_{i}}^{-u_{s}}d_{t_{i}}^{-u_{t}}}$$

where $d_{t_{i}}$ is the temporal distance and $u_{t}$ is the user-defined temporal distance-decay factor.

However, based on the weighting mechanism (Equation (14)), we can see that the function will introduce an error when either the spatial $\left(d_{s_{i}}\right)$ or the temporal $\left(d_{t_{i}}\right)$ distance is 0 (divided by zero error). Therefore, we need to specify the smallest distance possible in both the spatial and temporal dimension. For our experiment, we set the smallest spatial $\left(d_{s_{i}}\right)$ and temporal $\left(d_{t_{i}}\right)$ distance at 1. These settings are artificially chosen by the user based on the fact that the lowest possible spatial and temporal distance (excluding 0) in this dataset is 1.

#### 2.3.2. Distribution-Based Distance Weighting

**Figure 4:**
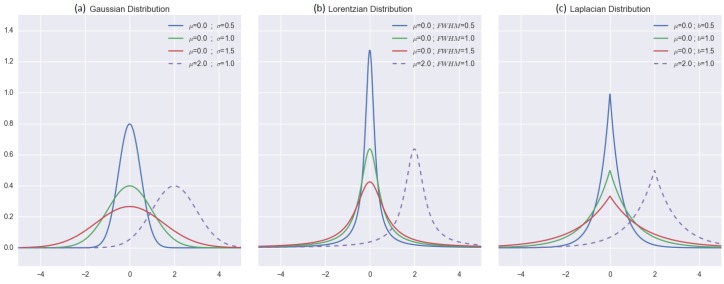
Distribution methods that are proposed in this work for comparisons: (**a**) Gaussian; (**b**) Lorentzian and (**c**) Laplacian distributions. Within the proposed DDW, the horizontal axis represents the distance, and the vertical axis is the weight values.

In this paper, we also introduce the so-called distribution-based distance weighting (DDW) technique. It considers nearby data variations (similar to improved IDW, as shown in Figure 3) to produce the distribution for the weight calculations. Below are the distribution equations that are used and compared in this work:

(15) $$Gaussian\left(x,\mu ,\sigma \right)=\frac{1}{\sqrt{2\pi }\sigma }\cdot e^{-\frac{{\left(x_{i}-\mu \right)}^{2}}{2\sigma^{2}}}$$

(16) $$Lorentzian\left(x,\mu ,\Gamma \right)=\frac{\Gamma }{2\pi }\cdot \frac{1}{x^{2}+{\left(\frac{\Gamma }{2}\right)}^{2}}$$

(17) $$Laplacian\left(x,\mu ,b\right)=\frac{1}{2b}\cdot e^{-\frac{\left|\mu -b\right|}{b}}$$

where *μ* is the mean of the data $D=\{v_{1},v_{2},v_{3},\ldots ,v_{i},\ldots ,v_{N}\}$. For each of the distributions: *σ* is the standard deviation for the Gaussian; Γ is the full width at half maxima (FWHM) for the Lorentzian; and *b* is the “diversity” (or scale parameter) for the Laplacian distribution (Figure 4). However, for the purpose of this work, we will regard Γ and *b* as equivalent to the standard deviation *σ*. The calculation for DDW is in the form:

(18) $$\hat{z}\left(x_{0}\right)=\sum_{i=1}^{N}\lambda {\left(d_{s_{i}}\right)}_{i}\cdot v_{i}$$

(19) $$\lambda {\left(d_{s_{i}}\right)}_{i}=Distribution\left(d_{s_{i}},\mu_{R},\sigma_{R}\right)$$

(20) $$\sigma_{R}=R\cdot \frac{\sigma }{max\{|min\left(D\right)-\mu_{D}|,|max\left(D\right)-\mu_{D}|\}}$$

where $Distribution(d_{s_{i}},\mu_{R},\sigma_{R})$ can be either a Gaussian, Lorentzian or Laplacian distribution with $\mu_{R}=0$ (the middle position).

The idea of this approach is that when the data *D* variations are very small (i.e., low *σ*), the distribution will have a fairly sharp peak and will cause the weighting to be more sensitive to the distance $\left(d_{s_{i}}\right)$. On the other hand, if the *σ* is high, this means that the values included in the calculation are more spread out. This makes the width of the “bell” very wide, which results in the $d_{s_{i}}$ having less impact on the weight calculation. This method is capable of reducing the “bull’s eye” effect in the final results, which is a major problem in the conventional IDW technique [1]. However, it is notable that our proposed conventional reduction approach of the STI technique is not designed for this purpose. This is because in some cases, a very smooth surface for geospatial analysis can be very unrealistic.

## 3. Simulation Procedure and Performance Assessment

### 3.1. Sensor Networks Spatial Samplings

This methodology will be used to obtain the near optimal placement of sensor nodes (Section 3.2) that would best represent (for a specific environmental parameter) the whole region of interest (ROI), based on a given fixed number of nodes (N).

For this purpose, we adopt Python’s scipy built-in module, the differential_evolution (DE) algorithm [21], to select the locations of observed points, instead of just randomising them. This procedure is implemented based on the fact that if we entirely randomising them, there is a possibility that the stochastically-picked location points would be clustered in a specific region, and no interpolation method will produce acceptable results, especially when the sample size is very small. The results will be demonstrated in Section 4.2.

Since we are using the South Esk hydrological modelled data, we can get access to significant environmental information about the area. However, for the purpose of this simulation, we will utilise the ROI’s elevation data from the dataset to calculate the fitness (or representativeness). Such a selection was made because a high-elevation sample is crucial in the dataset for meteorological studies [5].

This work is focusing on interpolation, which means that we have to manually pick 4 location points so that we could actually ‘interpolate’ the entire map; these are: topl-left, top-right, bottom-left and bottom-right. Then, the rest of location nodes (N−4) will become the individuals of the DE algorithm, represented as $X=\{x_{1},x_{2},\cdots ,x_{N}\}$. The objective of the optimisation is to maximise the leave-one-out cross-validation (LOCCV) error, so that extreme values (low and high elevation sample) are obtained [22]:

(21) $$LOOCV_{SSE}\left(X\right)=\sum_{i=1}^{N}\;{\left[\;IDW\left(x_{i},X_{i}\right)-o_{i}\;\right]}^{2}$$

where SSE denotes sum-squared-error; $IDW(x_{i},X_{i})$ represents the estimated value using IDW interpolator at location $x_{i}$ using the remaining nodes (such that $X_{i}=X\backslash \left\{x_{i}\right\}$); and $o_{i}$ is the observed valued at the *i*-th location. In addition, sparsity is an important aspect in sensor network deployment in a way that well-distributed nodes’ locations are preferred:

(22) sparsity(X)=σ(X)/μ(X)

where *σ* and *μ* are the calculated standard deviation and the mean value from the pair-wise distances in *X*. Finally, the optimisation process is designed to minimise the equation below to comply with the objectives:

(23) $$fitness=LOOCV_{SSE}{\left(X\right)}^{-1}\cdot sparsity\left(X\right)$$

### 3.2. Choosing a “Balanced” 2D Spatial Interpolation Method

First of all, we compare the computational efficiency of currently-available 2D spatial interpolation algorithms. Based on the assumption that we will collect a huge amount of data (up to thousands of items) with a very high temporal frequency (every second), the computational efficiency is a crucial factor in the algorithm selection. Therefore, we must select a ‘balanced’ algorithm that produces an acceptable result in a timely manner.

In this section, we will compare four methods: kriging, basic IDW, improved IDW and TIN. The comparison will be divided into two steps: (1) evaluate the total elapsed time of each method, and discard the method that requires an unacceptable time frame to process; (2) objectively compare the quality of the final output using root mean squared error (RMSE). RMSE is a frequently-used error measurement tool based on the difference between the estimated value produced by the interpolator, and the observed value; and it has been recognised as the best overall method for spatial analysis comparison [12]. It is in the form of:

(24) $$RMSE={\left[\frac{1}{N}\sum_{i=1}^{N}{\left(p_{i}-o_{i}\right)}^{2}\right]}^{\frac{1}{2}}$$

where *N* is the total number of cells and $p_{i}$ and $o_{i}$ are the *i*-th predicted and observed points, respectively. It should be also noted that the RMSE largely favours the largest error (i.e., $p_{i}-o_{i}$ in Equation (24)) to the detriment of the lowest terms; in addition, a different/larger exponent could also be chosen to exaggerate such phenomena.

Furthermore, in order to remove the "bias" of RMSE, where it favours more larger errors, we also utilised mean absolute error (MAE) and the corresponding standard error (SE) using:

(25) $$MAE=\frac{1}{N}\sum_{i=1}^{N}\left|p_{i}-o_{i}\right|$$

(26) $$SE=\frac{\sigma }{\sqrt{N}}$$

where *σ* represents the standard deviation of the list of error (i.e., calculated by $|p_{i}-o_{i}|$ as in Equation (25)).

In this experiment, we will compare the statistical quality and the computational efficiency of each method to choose a “balanced” spatial interpolation method for our STI algorithm to be developed at a later stage. The chosen method will be used for further comparisons in the following experiments.

### 3.3. Comparing Spatiotemporal Approaches and Evaluating the DDW

The fundamental idea of the STI method is to develop an algorithm to measure the environmental situation at an unmeasured time slice and to show how well the algorithm can reconstruct the “missing” time slices. This experiment is designed mainly for that purpose, as well as to define which temporal approach (extension or reduction) will produce better results.

**Figure 5:**
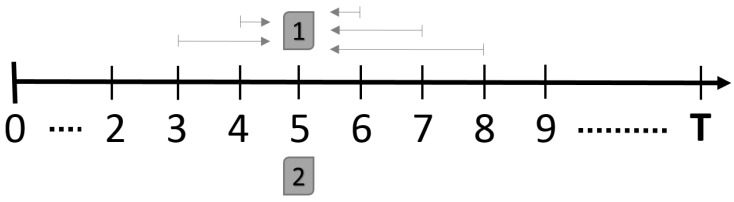
Demonstration of the entire simulation setup for each time slice using a single scheme. The example above interpolates Time Index 5, using scheme (2, 3). Map 1 (top) is the “estimated” map generated using the STI method; Map 2 (bottom) is the “observed” map based on the original data. The objective performance of the algorithm is measured using RMSE.

Our South Esk Hydrological model holds data in one-hour time intervals. For each time index, we reconstruct the map value using the proposed STI method using different schemes. The "scheme" refers to the previous and next window length to be included in the interpolation process. For example, we would like to interpolate Time Index 5 (Figure 5), and the scheme is (2, 3). This means that Time Indices 3, 4, 6, 7, 8 are used to estimate the map at Time Index 5. However, we skip the iterations that would result in an invalid time index value.

## 4. Results

### 4.1. Original Data Visualisation

**Figure 6:**
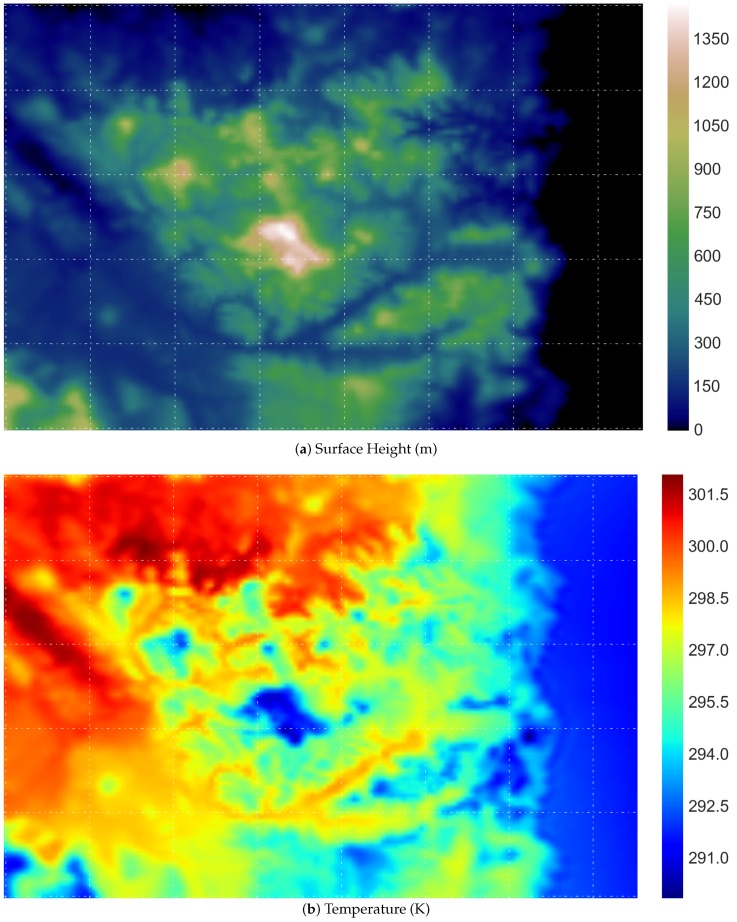
Visualisation of the South Esk hydrological modelled dataset used throughout the simulation: (**a**) the elevation map, in meters; and (**b**) the air temperature data, in Kelvin.

Figure 6 demonstrates a visualisation of the original data used in this work. The surface height data are mainly utilised for the purpose of selecting sensor location using an evolutionary algorithm (differential evolution, as described in Section 3.1); whilst the air temperature data are used for the rest of the simulations.

### 4.2. Comparing Spatial Interpolation Methods

**Figure 7:**
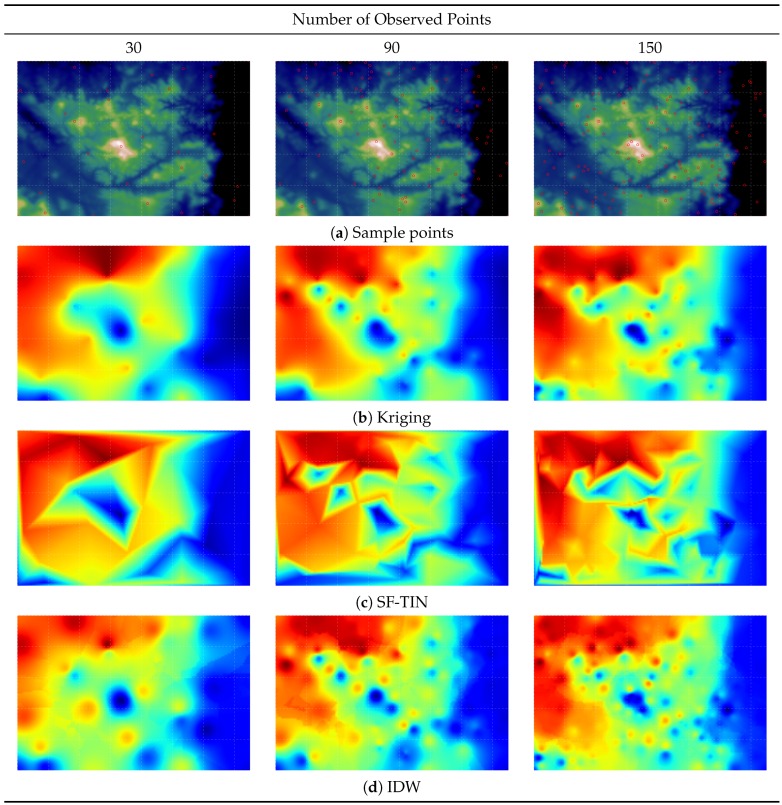
Visualisation output for: the selected sample point locations (**a**) using the optimisation technique (DE); and three widely-used different spatial interpolation techniques (**a**) ordinary kriging, (**b**) triangular irregular network and (**c**) inverse distance weighting], compared to different numbers of sample points (N=30,90,150).

Figure 7 is a demonstration of the maps that are generated by the interpolation techniques. We can see that kriging creates the smoothest map overall. TIN is visually abrupt, whereas IDW creates gradual visual results with an observable “bull’s eye” effect. The following experiment is designed to evaluate the computational efficiency of each method.

**Figure 8:**
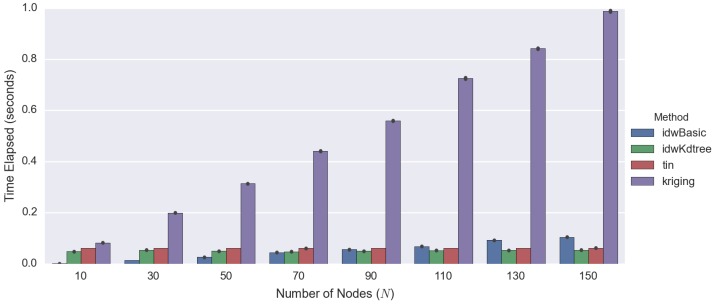
This graph demonstrates a comparison of computational efficiency for different 2D spatial interpolation techniques, with the data based on Table 3.

Based on Figure 8, kriging is the most computationally-intensive method compared to other techniques, irrespective of the number of observed points. The time required for kriging increases substantially as the number of observed values grows. Since computational efficiency is a crucial factor in this paper (Section 1.1) as previously mentioned, kriging-based spatiotemporal algorithms are not a suitable technique for our purpose and will not be considered and compared.

On the other hand, among the three different techniques compared (kriging, TIN and IDW), IDW performs best overall. If the number of observed points is small, basic IDW performs well. However, as the number of observed points increases, the processing time for basic IDW also increases linearly. It will reach a point where the improved IDW performs better than basic IDW (i.e., N=90 in Figure 8).

The time required for both TIN and the improved IDW is O(logn), which means that they are suitable for use with a large-scale problem. Then, we compare these techniques based on interpolation performance.

**Figure 9:**
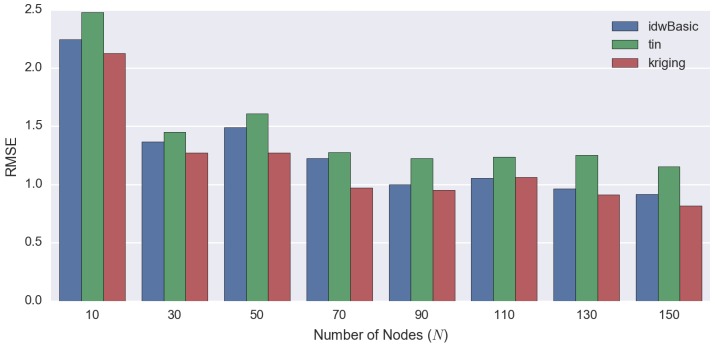
This graph demonstrates the quantitative comparisons (RMSE) between the different 2D spatial interpolation techniques, using the data as shown in Table 4.

**Figure 10:**
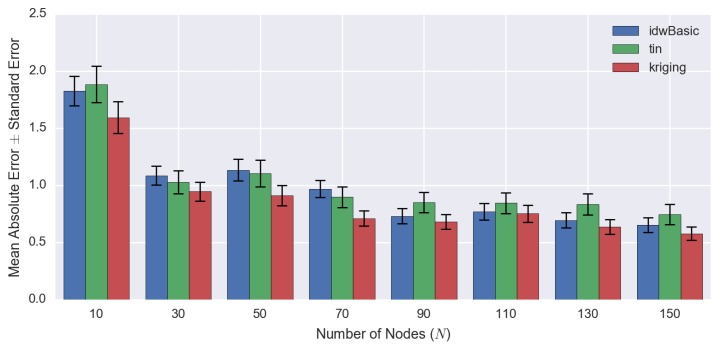
This graph demonstrates the MAE with the corresponding SE between the different 2D spatial interpolation techniques, using the data as shown in Table 4.

Based on Figure 9 and Figure 10, the statistical error decreases gradually as the number of observed values (*N*) increases. It is obvious that kriging produces the best objective results amongst the spatial interpolation techniques (lowest RMSE) regardless of *N*, but it is very computationally intensive. On the other hand, IDW and TIN have comparable statistical error. However, it is interesting to see that for some cases (i.e., when N={30,50,70}), the MAE of IDW is higher than TIN, but its RMSE is lower. The occurrence of this phenomenon indicates that IDW generates more error than TIN on average, but it also signifies that the error being produced by TIN is more extreme resulting from the ‘bias’ of RMSE (as previously discussed in Section 3.2).

### 4.3. Comparing Spatiotemporal Approaches

In this experiment, we will compare the performance of two approaches to temporal interpolation: extension and reduction [14]; and we will utilise the improved IDW method, which is the most suitable spatial interpolation technique according to the previous results (Section 4.2, Comparing Spatial Interpolation Methods).

**Figure 11:**
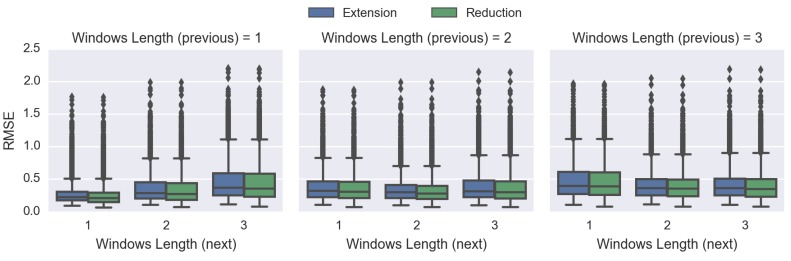
Performance comparisons between different temporal interpolation approaches using the improved IDW: extension (existing) and reduction (proposed) approaches. The vertical axis is the RMSE of the respective (previous and next) windows’ length.

Figure 11 shows that whenever the scheme (either previous or next) is zero, it leads to very poor performance. However, this kind of scheme is actually extrapolating instead of interpolating, which is outside of the scope of this work. Therefore, we will not make any conclusions based on these results.

For each of the schemes, the extension approach always performs more poorly than the reduction method (i.e., higher RMSE). These results confirm that our proposed reduction approach is superior to the current extension approach and is more suitable as the STI technique.

**Figure 12:**
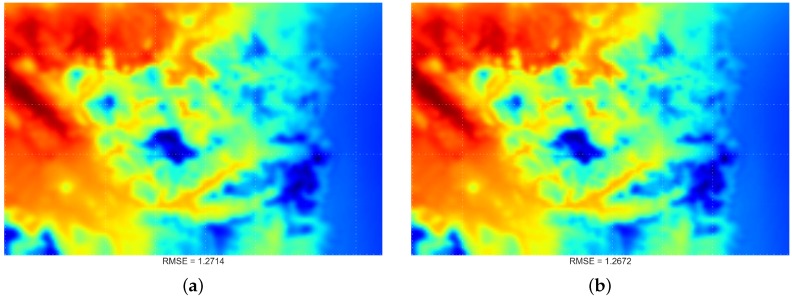
Visualisation output for the comparison results between the extension- and reduction-based STI methods. In this output, we used the scheme involving plus-and-minus one time index. This selection is based on the results obtained from Figure 11, which shows such a configuration produces the best results overall (lowest mean RMSE). (**a**) Extension approach; (**b**) reduction approach.

Lastly, Figure 11 indicates that the scheme of including one “previous” and one “next” time index will produce the lowest RMSE overall, and a visualisation demonstration is provided in Figure 12. Such a configuration will be used in the following experiments.

### 4.4. Comparing the Proposed DDW Methods

**Figure 13:**
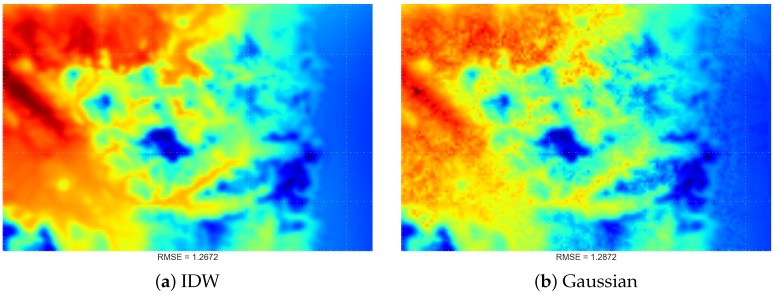

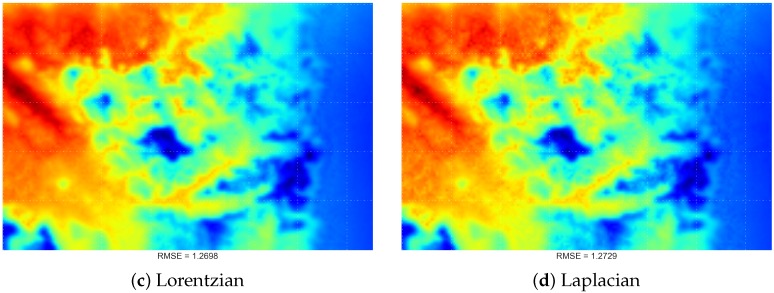
Visualisation output of IDW (**a**) and the proposed DDW: (**b**) Gaussian; (**c**) Lorentzian; and (**d**) Laplacian; using the proposed spatiotemporal interpolation technique.

In this experiment, we compared the IDW method with the proposed DDW method. In Figure 13, there is little visual difference between STI techniques using either IDW or the proposed DDW methods. However, in general, DDW methods are more likely to produce smoother surfaces than the IDW technique.

**Figure 14:**
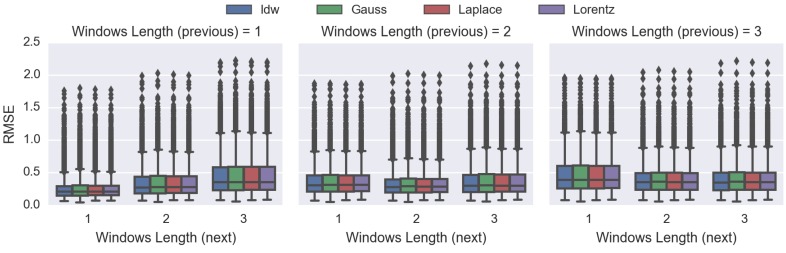
Objective comparisons between the IDW method and the proposed DDW techniques: using Gaussian, Lorentzian and Laplacian distributions. This graph demonstrates the RMSE comparison between the respective (previous and next) windows’ length.

The results in Figure 14 show that IDW is superior to DDW. On average, the RMSE of IDW is approximately 38% lower than that of the DDW techniques, which is a significant difference. In this experiment, the graph also suggests that using the scheme of plus-and-minus one time index will produce the lowest error overall.

## 5. Discussion

We summarise the strengths and weaknesses of the 2D spatial interpolation techniques in Table 5. The kriging interpolation technique performs quantitatively best overall (Table 4 and Figure 9). However, the time required for processing increases dramatically as the number of observed points grows (Figure 8). Thus, because of the fact that computational efficiency is a crucial factor in our work, kriging is the least preferred method.

The improved IDW technique that utilises the kd-tree algorithm and the TIN technique using Delaunay triangulation have comparable computational efficiency O(logn) and similar objective performance. Nevertheless, from an environmental manager’s perspective, visual continuity is an important factor in making a confident interpretation. Because the TIN method is visually abrupt (Figure 7), it is less suitable for environmental applications [2], and so, IDW is considered the most suitable spatial interpolation method overall.

Figure 11 and Figure 14 demonstrate that whenever the previous or the next time index to be included is zero, the RMSE goes up significantly, leading to a poor performance. The reason is that when we do not include any of the previous or the next time index, we are actually extrapolating rather than interpolating that particular time frame, which is outside the scope of this work.

The results in Figure 11 indicate that our proposed IDW-based reduction spatiotemporal interpolation approach is superior to the currently-available extension temporal method. Consequently, the reduction temporal approach will be utilised. Furthermore, the optimal time frame to be included in the STI is plus-and-minus one time index, and this will yield the best results overall (Figure 11 and Figure 14).

Up until now, we have concluded that a combination of the improved IDW technique using the reduction approach for STI would lead to a balanced result overall, in terms of computational efficiency and objective performance. In this work, we evaluated the performance of our proposed DDW-based STI (Figure 14). However, the proposed DDW showed very little benefit over IDW in terms of RMSE.

Finally, one major advantage of our proposed reduction approach over the extension approach is that the user does not need to justify the temporal factor (*c*) as in Equation 12, which is always done manually and is highly dependent on the user’s expertise.

## 6. Conclusions and Future Work

Our study shows that the improved IDW spatial interpolation algorithm, combined with the reduction approach for temporal interpolation, was found to be the most effective technique overall for spatio-temporal interpolation (STI) within environmental modelling applications. This technique provided the following advantages over the alternative STI techniques investigated:

1. Computational time does not increase as the number of sample points grows significantly;
2. The objective results are comparable with other techniques;
3. The technique does not create abrupt visual results.

The results obtained from this work are only applicable for gap filling rather than interpolating over a longer term basis where climate variation patterns need to be considered, e.g., interpolating daily temperature data for consecutive days. This issue will be addressed as a further research direction.

The distribution-based distance weighted (DDW) STI technique, also proposed in this paper, did not perform as well as the inverse distance-weighted (IDW) method and so is not recommended for environmental modelling applications.

The proposed method in this paper has a broad potential of applicability within environmental research, such as oceanography, geography, hydrology, and so on. Since there is no optimal STI interpolation method that is suitable for every kind of problem, our proposed method could perform better in some cases.

The current work relies in its results and conclusions on the demonstration conducted on real data. Therefore, the findings are limited to this set of data and cannot be generalized unless a more rigorous approach is conducted. It is recommended as future work that a theoretical demonstration be performed.

While kriging has not been applied for our spatio-temporal interpolation, as the focus on the current work was computational efficiency, we strongly encourage the use of this technique when accuracy is a major concern ([23] and the references therein).

## Figures and Tables

**Table 1:** List of previous studies that utilised different interpolation techniques for environmental studies, and the findings from each study are also provided. Comparing methods as done here cannot be generalized, as this is limited to the empirical exercises, and no rigorous theoretical demonstration is provided (the table is sorted based on the similarity of interpolation techniques adopted and compared in each study).

Process	Techniques	Findings	Ref.
Spatial rainfall mapping using IDW in the middle of Taiwan.	IDW	- IDW can be improved by the adjustment of the distance-decay parameter and the search radius.	[4]
		- Does not have significant interpolating ability for extreme values.	
Compared to the developed CAR method with IDW for climate datasets in China.	IDW, CAR	- High-elevation data are an important factor for meteorological studies.	[5]
		- CAR performs slightly better than GIDW in terms of objective comparisons, especially in estimating local neighbouring patterns.	
Comparing different techniques for precipitation and elevation.	IDW, AIDW, Kriging	- Varying the distance-decay parameter of IDW based on the spatial pattern can improve overall performance (AIDW).	[6]
		- AIDW can perform better than kriging in some cases.	
Proposing regression-based IDW and comparing it with IDW and kriging.	IDW, RIDW, Kriging	- Integrating linear regression in IDW provides comparable objective evaluation to kriging and is computationally less demanding.	[7]
		- The confidence interval (CI) of RIDW also surpasses kriging CI.	
Evaluating different interpolation techniques for air temperature data.	Spline, IDW, Kriging	- Kriging performs better overall, followed by IDW and spline.	[8]
Comparing different techniques for rainfall mapping in Sri Lanka.	IDW, TPS, OK, BK	- Interpolation results are very much dependent on the settings.	[9]
		- Different methods with different settings must be tested to define the suitable technique.	
		- Bayesian kriging and splines performed best overall.	
Assessing different interpolation methods to define the most suitable technique for the McArthur Forest Fire Danger Index (FFDI).	IDW, OK, RF, RFOK, RFIDW	- Combination of methods: RFOK and RFIDW shows the most promising results (least error).	[1]
		- Fire danger index is highly related to the behaviour of climate change, and should be considered carefully.	
Investigated several techniques for depth to underground water in northwest China.	IDW, RBF, OK, SK, UK	- Simple kriging is the optimal method in terms of result consistency and the smallest prediction interval of 95%.	[10]
		- Depth of underground water increases significantly over the year because of excessive exploitation.	
Ranking spatial interpolation techniques using GIS-based DSS.	Spline, IDW, Kriging, TP, TS	- No optimal technique that can accurately predict the rainfall.	[11]
		- Performance of each technique depends on the scale of the input data.	
		- Kriging is the recommended technique, as it produces the most consistent results.	
Using an interpolation method to construct a comprehensive archive of Australian climate data.	TPS, OK	- Different climate variables can be more accurately interpolated using different techniques due to the characteristic variability.	[3]

**Table 2:** Abbreviation list of the interpolation techniques used in Table 1.

Title	Abbreviations
IDW	Inverse Distance Weighting
AIDW	Angular IDW
GIDW	Gradient IDW
SK	Simple Kriging
OK	Ordinary Kriging
BK	Bayesian Kriging
UK	Universal Kriging
CAR	Clustering Assisted Regression
TPS	Thin Plate Splines
RBF	Radial Basis Function
RF	Random Forest
RFIDW	RF + IDW
RFOK	RF + OK
TS	Trend Surface
TP	Thiessen Polygon

**Table 3:** Total normalised time elapsed for each method (0: fastest, 1: slowest).

Number of Observed Points	Total Time Elapsed			
	Basic IDW	Improved IDW	TIN	Kriging
10	0.0024	0.0252	0.0289	0.3034
30	0.0096	0.0278	0.0281	0.3852
50	0.0214	0.0268	0.0291	0.4706
70	0.0355	0.0253	0.0291	0.6582
90	0.0447	0.0040	0.0286	0.7808
110	0.0545	0.0283	0.0288	0.8633
130	0.0596	0.0322	0.0283	0.9720
150	0.0596	0.0322	0.0283	0.9720

**Table 4:** Statistical error (RMSE and MAE with its corresponding SE) for each method. In this graph, we only show one ‘IDW’, because the errors of both versions of IDW (basic and improved, based on Table 3) are the same; the only difference between the two being the processing time.

Number of Observed Points	RMSE			MAE ± SE		
	IDW	TIN	Kriging	IDW	TIN	Kriging
10	2.2461	2.4767	2.1257	1.83 ± 0.13	1.89 ± 0.16	1.60 ± 0.14
30	1.3667	1.4503	1.2720	1.09 ± 0.08	1.03 ± 0.10	0.95 ± 0.08
50	1.4897	1.6080	1.2718	1.14 ± 0.10	1.11 ± 0.12	0.91 ± 0.09
70	1.2259	1.2755	0.9727	0.97 ± 0.07	0.90 ± 0.09	0.71 ± 0.07
90	0.9985	1.2243	0.9517	0.73 ± 0.07	0.85 ± 0.09	0.68 ± 0.07
110	1.0565	1.2378	1.0640	0.77 ± 0.07	0.85 ± 0.09	0.75 ± 0.07
130	0.9645	1.2533	0.9136	0.70 ± 0.07	0.84 ± 0.09	0.64 ± 0.07
150	0.9146	1.1537	0.8168	0.65 ± 0.06	0.75 ± 0.09	0.58 ± 0.06

**Table 5:** Summary of the strengths and weaknesses of the spatial interpolation methods evaluated in this paper: ordinary kriging (OK), inverse distance weighting (IDW) and triangle irregular network (TIN). These evaluations are based on three criteria: objective performance (RMSE), computational efficiency and the final visual output smoothness.

	Advantages	Disadvantages
OK	- Geostatistical method that considers spatial pattern. - Best objective comparison overall. - Creates very smooth surface.	- Computationally very expensive. - Unsuitable for very large datasets.
IDW	- Computationally inexpensive. - Can be improved using kd-tree to increase computational efficiency. - Creates smooth surface.	- Produces “bull’s eye effec” for extreme values. - Unsuitable for large scale problems without using kd-tree algorithm.
TIN	- Computationally inexpensive.	- Produces abrupt surfaces.

## Abbreviations

The following abbreviations are used in this manuscript:

ROI	Region of interest
STI	Spatio temporal interpolation
DDW	Distribution-based distance weighting
IDW	Inverse distance weighting
AIDW	Angular IDW
GIDW	Gradient IDW
SK	Simple kriging
OK	Ordinary kriging
BK	Bayesian kriging
UK	Universal kriging
CAR	Clustering assisted regression
TPS	Thin plate splines
RBF	Radial basis function
RF	Random forest
RFIDW	RF + IDW
RFOK	RF + OK
TS	Trend surface
TP	Thiessen polygon
RMSE	Root mean squared error
MAE	Mean absolute error
SE	Standard error
